# Discovery of Surfactant-Like Peptides from a Phage-Displayed Peptide Library

**DOI:** 10.3390/v12121442

**Published:** 2020-12-15

**Authors:** Toshiki Sawada, Rina Oyama, Michihiro Tanaka, Takeshi Serizawa

**Affiliations:** 1 Department of Chemical Science and Engineering, School of Materials and Chemical Technology, Tokyo Institute of Technology, 2-12-1-H121 Ookayama, Meguro-ku, Tokyo 152-8550, Japan; rooyama@polymer.titech.ac.jp (R.O.); mtanaka@polymer.titech.ac.jp (M.T.); 2 Precursory Research for Embryonic Science and Technology, Japan Science and Technology Agency, 4-1-8 Honcho, Kawaguchi-shi, Saitama 332-0012, Japan

**Keywords:** peptide, phage display, emulsion, surface activity, screening

## Abstract

Peptides with specific affinities for various materials have been identified in the past three decades and utilized in materials science and engineering. A peptide’s capability to specifically interact with materials is not naturally derived but screened from a biologically constructed peptide library displayed on phages or cells. To date, due to limitations in the screening procedure, the function of screened peptides has been primarily limited to the affinity for target materials. Herein, we demonstrated the screening of surfactant-like peptides from a phage-displayed peptide library. A screened phage clone displaying a peptide showed high activity for accumulating at emulsion surfaces with certain assembled structures, resulting in stable emulsions. The surface tension for the solution of the chemically synthesized peptide decreased with increasing peptide concentration, demonstrating certain surface activity, which corresponded to the ability to decrease the surface tension of liquids (e.g., water), owing to the accumulation of molecules at the air–liquid or liquid–liquid interface. Peptides with a randomized sequence did not lower the surface tension, indicating the essential role of amino acid sequences in surface activity. Our strategy for identifying novel functional peptides from a phage-displayed peptide library can be used to expand the applicability of peptidyl materials and biosurfactants.

## 1. Introduction

Over the past three decades, novel peptides with non-naturally determined functions, such as specific affinity for artificial materials, have been successfully developed [1,2]. The structures of unique peptides (e.g., amino acid components, sequences, secondary structures) were not rationally designed to obtain the functions but were screened from a biologically constructed peptide library displayed on bacteriophages (phages) or cell surfaces [1,3]. To date, various materials have been applied to peptide targets, and the resultant peptides show specific affinities for the material. These peptides have been considered to recognize the atomically or molecularly ordered three-dimensional structures of the materials’ surfaces [4] and utilized as molecular tools for functionalization of materials [1,5]. Due to the large molecular number of peptides in biologically constructed libraries, peptides with various functions other than specific affinity should be contained within such libraries; in practice, however, the design of the screening procedure has been limited to the identification of “affinity peptides” because of limitations in the screening process, which requires the isolation of evolved peptides from others. Several peptides with biomineralization capabilities to mediate inorganic crystal formation were incidentally discovered through the affinity-based screening procedure because the peptides enabled capping of specific crystalline faces of the target materials [6,7,8,9]. Recently, catalytic-directed screening procedures for peptides using precipitation- or membrane separation-based isolation strategies have been reported [10,11,12]; therefore, screening design will widen the applicability of screening to obtain further artificially functionalized peptides.

Surfactants are a unique class of surface-active molecules that enable the dispersion of two immiscible liquids within a continuous liquid phase in the form of droplets [13]. These molecules are essential components in diverse applications in the pharmaceutical, food, and cosmetic industries [14,15,16]. Because synthetic surfactants, which are most often used as components for emulsion formation, show toxic and irritant properties [17], the biocompatibility of biopolymers such as native and/or denatured proteins makes them attractive surfactants to form emulsions compared with synthetic surfactants [18,19,20]. Such surfactant-like proteins can be classified into two distinct categories on the basis of their interfacial adsorption behavior: (1) the majority of surfactant-like proteins, which adsorb onto emulsion surfaces undergoing several degrees of denaturation [21,22], and (2) hydrophobins, the second class of surfactant-like proteins, which adsorb onto emulsion surfaces with native conformations [23]. Hydrophobins are a group of small (≈100 amino acids) proteins that exhibit the highest surface activity among all known proteins [24]. Hydrophobins preferentially adsorb to air–water interfaces compared to other biopolymers without conformational changes, indicating that their compact and robust tertiary structures can result in high surface activity.

**Figure 1:**
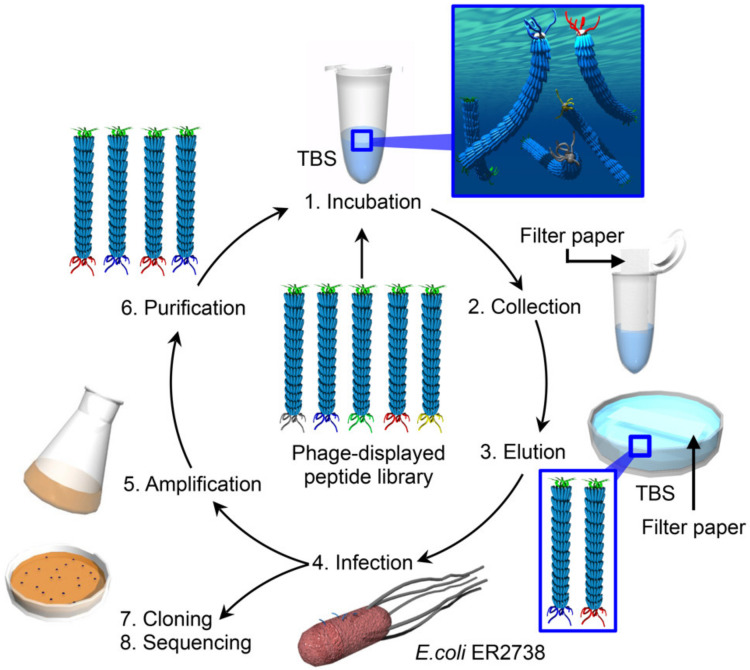
Schematic illustration of the screening of phage clones displaying peptides with an accumulation capability at air–liquid interfaces.

Peptides possess the natural physicochemical properties of proteins but have smaller molecular weights. Because peptide-based functional molecules are superior from the viewpoint of the flexibility of design and synthesis for modulating molecular assembly and introducing functionality [25,26,27,28,29,30], peptide surfactants are anticipated for drug delivery and related applications. However, the artificial design of peptides with high surface activity is challenging due to the control of their suitable amphiphilicity in terms of molecular design; therefore, the design of de novo peptide-based surfactants has focused on mimicking proteins with flexible backbone conformations [31,32,33] or hydrophobins with rigid backbone structures [34]. Here, we demonstrate an alternative strategy to obtain surfactant-like peptides by screening technology using a phage-displayed peptide library (Figure 1). Phage-displayed peptides with an ability to accumulate at air–liquid interfaces were successfully obtained by screening. Mixing of the screened phage clone aqueous solutions with an organic solvent resulted in the formation of stable oil-in-water emulsions, and the stability was greater than that obtained using wild type (WT) phages under suitable conditions. The phage clone formed different assembled structures at the emulsion surface compared to WT phages, possibly due to different surface activities. Surface tension measurements using pendant drops revealed a higher surface tension for the chemically synthesized identified peptide compared to the randomized peptide, showing the essential role of the amino acid sequence in the surface activity. Furthermore, the peptide was applied to emulsion formation and showed higher stability than that prepared with the randomized peptide, even though various organic solvents were used. These results revealed that our strategy for using M13 phages to screen peptides that accumulate at the air–liquid interfaces for the construction of surfactant-like peptides will open up an opportunity to discover novel peptide surfactants for use in a novel class of functional soft materials based on surfactants.

## 2. Materials and Methods

### 2.1. Materials

A Ph.D-12™ Phage Display Peptide Library Kit, a Ph.D. Peptide Display Cloning System, and *Escherichia coli* strain ER2738 were purchased from New England Biolabs (Ipswich, MA, USA). NovaSynTGR resin, 9-fluorenylmethyloxycarbonyl (Fmoc) amino acid derivatives, and 2-(1*H*-benzotriazole-1-yl)-1,1,3,3-tetramethyluronium hexafluorophosphate were purchased from Novabiochem. 1-Hydroxybenzotriazole monohydrate and *N,N’*-tetramethylenebismaleimide were purchased from Tokyo Chemical Industry Corporation (Tokyo, Japan). All other reagents were purchased from Nacalai Tesque (Kyoto, Japan). Ultrapure water with a resistivity of more than 18.2 MΩ·cm was supplied by a Milli-Q system (Merck Millipore, Burlington, MA, USA) and used for all the experiments.

### 2.2. Screening of Surfactant-Like Peptides from a Phage-Displayed Peptide Library

An M13 phage library containing 1.2 × 10^10^ plaque-forming units (pfu) of phage solution in 100 µL of Tris-buffered saline (TBS; 50 mM Tris, 150 mM NaCl, pH 7.5) was incubated for 30 min at ambient temperature. Filter paper (Advantec, Tokyo, Japan, cut with a size of 5 mm × 5 mm) was gently placed in contact with the air–liquid interfaces to collect the phages accumulated at the interfaces. Then, the filter paper was immersed in 100 µL of TBS to elute the phages. The eluted phages were amplified by infection with *Escherichia coli* strain ER2738, and the amplified phages were then purified using a polyethylene glycol/NaCl solution for use in the next round of peptide screening. Five rounds of peptide screening were repeated, followed by cloning and DNA sequencing of the phages.

### 2.3. Preparation of Phage-Based Emulsions

WT M13 phage was expressed using the Ph.D. Peptide Display Cloning System without any modification of DNA. A total of 400 µL of aqueous M13 phage solution (15 or 150 nM) was mixed with 400 µL of toluene followed by shaking the mixture by hand for 30 s according to a previously reported method [35]. The prepared emulsions were incubated at 25 °C. The emulsion size was determined by utilizing ImageJ software (version 1.52, developed at the National Institutes of Health, Bethesda, MD, USA) using a photograph taken by a digital microscope (QX800HD 720P 3D, AsOne, Osaka, Japan).

### 2.4. Atomic Force Microscopy (AFM) Observations

The morphology of the assembled structures at the emulsion surfaces was imaged by AFM (SPM-9700HT, Shimadzu, Kyoto, Japan) in tapping mode in air at ambient temperature. The emulsions were dispersed in distilled water to remove the freed M13 phage, and 2 µL aliquots of the diluted dispersion were deposited onto a freshly cleaved mica substrate and dried for at least 6 h at ambient temperature in a desiccator with silica gel.

### 2.5. Peptide Synthesis

Peptides with a free *N*-terminus and an amidated *C*-terminus were prepared by solid-phase peptide synthesis using standard Fmoc-based procedures according to a previously published protocol [36]. The peptide chains were assembled on a NovaSynTGR resin (amino group 0.25 mmol /g) using Fmoc amino acid derivatives (3 equiv. for amino group) with 2-(1*H*-benzotriazole-1-yl)-1,1,3,3-tetramethyluronium hexafluorophosphate (3 equiv. for amino group), 1-hydroxybenzotriazole monohydrate (3 equiv. for amino group), and *N*,*N*-diisopropylethylamine (6 equiv.) in *N*-methylpyrrolidone (NMP) for coupling and 20% piperidine in NMP for Fmoc group removal. To cleave the peptides from the resin and to remove the side chain protecting groups, we treated the resins with trifluoroacetic acid (TFA)/thioanisole/*m*-cresol (10/0.75/0.25, v/v/v) for 3 h. The crude peptides were purified by reverse-phase high-performance liquid chromatography (ELITE LaChrom, HITACHI High-Technologies, Tokyo, Japan) using a C18 column (COSMOSIL 5C18-AR-300, 20 × 150 mm, Nacalai Tesque) with a linear gradient from 99.9% H_2_O/0.1% TFA to 99.9% acetonitrile/0.1% TFA at a flow rate of 6 mL/min. The peptides were identified by liquid chromatography–mass spectrometry (Prominence UFLC system, MS-2020, Shimadzu).

### 2.6. Structural Characterization of Peptides

The circular dichroism (CD) spectra for the peptide dissolved in phosphate-buffered solutions (10 mM phosphate, pH 7.5) or a buffer containing 50 vol% trifluoroethanol (TFE) at a concentration of 100 µM were recorded using a CD spectrometer (J-725, JASCO, Tokyo, Japan) under the N_2_ atmosphere at 25 °C using a quartz cell with a thickness of 0.2 cm. The data represent the average of 4 scans in the wavelength range of 190-260 nm with a resolution of 0.5 nm and a scanning speed of 50 nm/min. Molecular mechanics (MM) calculations for the peptides were performed using MM2 (PerkinElmer, Waltham, MA, USA) and started from the α-helical structures.

### 2.7. Surface Tension Measurements

Pendant drop tensiometry was conducted using a Phoenix-Smart P-60 instrument (Meiwafosis, Tokyo, Japan) to determine the surface tension of the peptide solutions. A droplet (≈10 μL) containing the peptides (1 mg/mL, 5 mg/mL, or 10 mg/mL) was extruded from a 22-G needle in air. The surface tension was calculated by fitting the droplet shape to the Young–Laplace equation.

### 2.8. Preparation of Peptide-Based Emulsions

Four hundred microliters of organic solvents (toluene, hexane, or cyclohexane) was mounted onto the aqueous peptide solution (1 mg/mL, 400 µL). Four hundred microliters of the aqueous peptide solution was mounted onto 400 µL of chloroform. The solutions were applied to the sonication procedure using a microtip on a Sonifier 250 (Branson, Danbury, CT, USA) to mix for 30 s. The prepared emulsions were incubated at 25 °C. The emulsion volume was determined by ImageJ software using the coverage area of the photograph taken from the bottom by a standard digital camera.

## 3. Results and Discussion

Phage clones displaying peptides with an accumulating capability at the air–liquid interfaces were screened from a 12-mer phage-displayed peptide library. After five rounds of the screening procedure, peptides displayed on phage clones were identified (Table 1). Six different sequences (12c01-12c06) were determined from 26 randomly picked phage clones. The sequence of the peptide displayed on the 12c01 phage clone (SQDIRTWNGTRS) appeared 16 times, suggesting a high accumulating capability for the sequence (see below). The percent appearances for amino acids used in phage clones were compared with those in the library phage (Figure S1). The percent appearances for the specific amino acids were increased (e.g., Trp for hydrophobic and Arg for hydrophilic amino acids) and decreased (e.g., Leu, Ala, Val, Phe, Tyr, and Met for hydrophobic amino acids and His and Lys for hydrophilic amino acids). Pro, which contributed to rigidity without secondary structural formation to peptides and proteins due to the kink ring structure, was decreased, indicating flexible features to form certain secondary structures of the accumulated peptides (see below). These observations suggest that the library phage was directed to specific phage clones.

**Figure 2:**
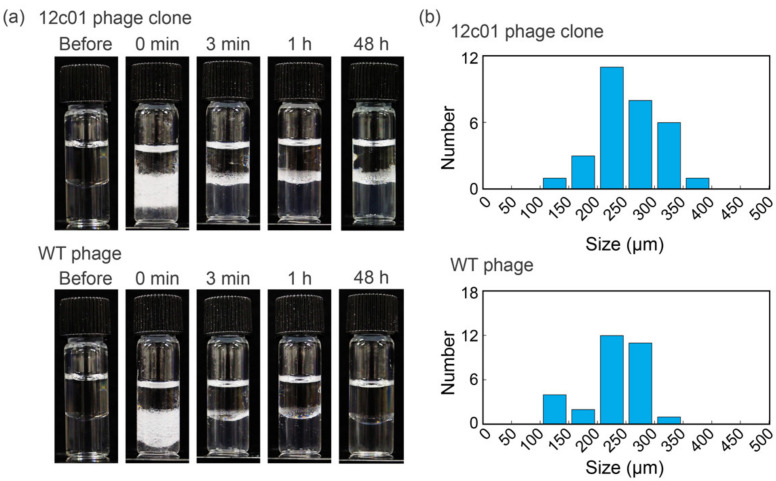
(**a**) Optical photographs of emulsions formed with the 12c01 phage clone and wild type (WT) phages (each 15 nM). (**b**) The size distribution for the emulsion droplets prepared with the 12c01 phage clone and WT phages immediately after emulsification.

The surface activity of the 12c01 phage clone was characterized through emulsion formation. Phage-based emulsions were produced using toluene as the oil phase (Figure 2a). Immediately after emulsification, the emulsion volume prepared using the 12c01 phage clone was almost the same as that prepared using WT phages when 15 nM M13 phage was used for emulsification. Rapid creaming within several minutes was observed for both the 12c01 phage clone and WT phages. Significantly, the emulsion using the 12c01 phage clone was maintained for at least 48 h, whereas that using WT phages was almost demulsified 3 min after emulsification, indicating a higher accumulated capability of the 12c01 phage clone to stabilize emulsions. The results for the differences in emulsion stability demonstrated that the 12c01 phage clone had higher surface activity than WT phages. We further measured the sizes of at least 50 different emulsion droplets stabilized by the 12c01 phage clone or WT phages immediately after emulsification for statistical analyses of the distribution (Figure 2b). The average sizes and distributions of emulsion droplets immediately after emulsification were the same within experimental error (257 ± 57 µm and 225 ± 48 µm for the 12c01 phage clone and WT phages, respectively), indicating that the creaming speed and/or the Ostwald ripening effects did not play essential roles in the high stability of the 12c01 phage clone-based emulsion. In other words, the peptide displayed on the 12c01 phage clone affected the stability after emulsion formation.

**Figure 3:**
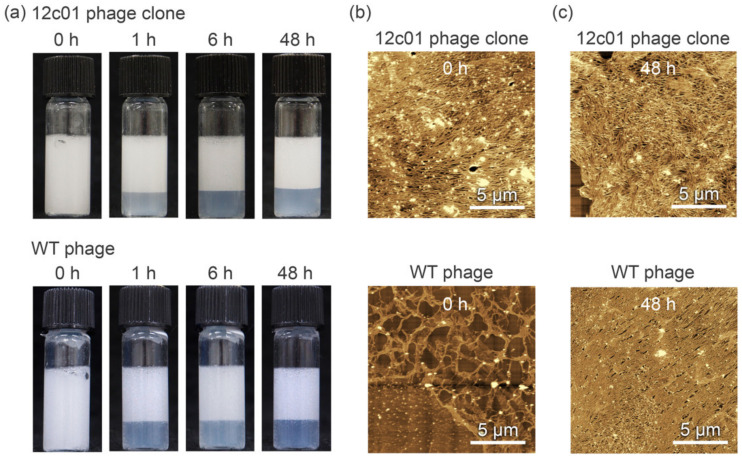
(**a**) Optical photographs of emulsions formed with the 12c01 phage clone and WT phages (each 150 nM). (**b**,**c**) Morphological characterization of the emulsion droplet surfaces. The emulsions prepared with the 12c01 phage clone and WT phages were observed immediately after emulsification (**b**) and 48 h later (**c**).

When a higher concentration of phage (150 nM) was used for emulsification (Figure 3a), the stability of the emulsions prepared with the 12c01 phage clone and WT phages was almost the same as that after 48 h of incubation due to the sufficiently high stability of the WT phage-based emulsion, as we have reported previously [35]. AFM was performed to characterize the assembled M13 phage structures at the emulsion interfaces (Figure 3b,c). Immediately after emulsification, fibrous structures of several micrometers in size, which are longer than that of the original phage (approximately 1 µm), were observed at the interface of emulsions prepared using the 12c01 phage clone and WT phages. Importantly, the surface coverage of emulsion droplets prepared by the 12c01 phage clone was higher than that prepared by WT phages, indicating certain effects of the displaying peptide on emulsion formation. After sufficient incubation to form a stable emulsion (48 h), the surface coverages for the droplets prepared with the 12c01 phage clone and WT phages were almost the same, even though the assembled structures were different (Figure 3c). Namely, although well-aligned ordered fibrous structures were observed in the emulsion droplets prepared with WT phages, the 12c01 phage clone formed less-ordered structures (Figure S2). We have previously found that WT phages formed ordered microstructures at “thermodynamic equilibrium” with structural relaxation during incubation when fewer fibrous structures were covered immediately after emulsification at a lower concentration of WT phages (150 nM), whereas “kinetically trapped” less-ordered structures were formed at a higher concentration of WT phages (1500 nM) [35]. Thus, higher surface coverage of the 12c01 phage clone (that is, higher amounts of fibrous structures) immediately after emulsification can result in kinetically trapped less-ordered structures after sufficient incubation, even though the 12c01 phage clone is used at a lower concentration (150 nM). These results indicate that the peptide-displaying 12c01 phage clone enables high surface activity to sufficiently cover emulsion droplets immediately after emulsification and affects the self-assembled structures at the emulsion surface to stabilize emulsions.

In the previous paragraphs, we showed the higher surface activity of the peptide-displaying M13 phage (that is, 12c01 phage clone) compared to WT phages. These results suggest that the dodecapeptide displayed on the 12c01 phage clone (12c01 peptide) had an accumulation capability at the air–liquid interfaces. Although the evaluation of phage clones can enable one to estimate the capability of the peptide displayed on the phage clones, direct evaluation of peptide is difficult due to the huge molecular weight of phages, plural display of peptides on phages, and formation of suitable conformations on phages [37,38,39,40,41]. Therefore, it is necessary to evaluate the accumulation of peptides free from phages. Thus, the chemically synthesized 12c01 peptide was used for further experiments. CD spectra for the 12c01 peptide (Figure S3) revealed negative Cotton effects at 222 nm and 198 nm in a buffer solution, which were assigned to the α-helix and random coil conformations, respectively. The α-helical content of the peptide increased in the TFE-containing buffer solution, and the peptide had the potential to adapt to the α-helix conformations in a hydrophobic atmosphere. The results from MM calculations for the 12c01 peptide with the α-helix conformation indicated that the lateral groups of hydrophobic residues (Ile and Trp) were located at a similar face (Figure S4a), possibly resulting in surface activity owing to the amphiphilic property. A control peptide with a randomized amino acid sequence for the 12c01 peptide (ran-12c01, sequence: IQNTRGDSTRSW) was newly designed and synthesized. MM calculations indicated that the hydrophobic Ile and Trp residues in the ran-12c01 peptide were not located at a similar face (Figure S4b).

**Figure 4:**
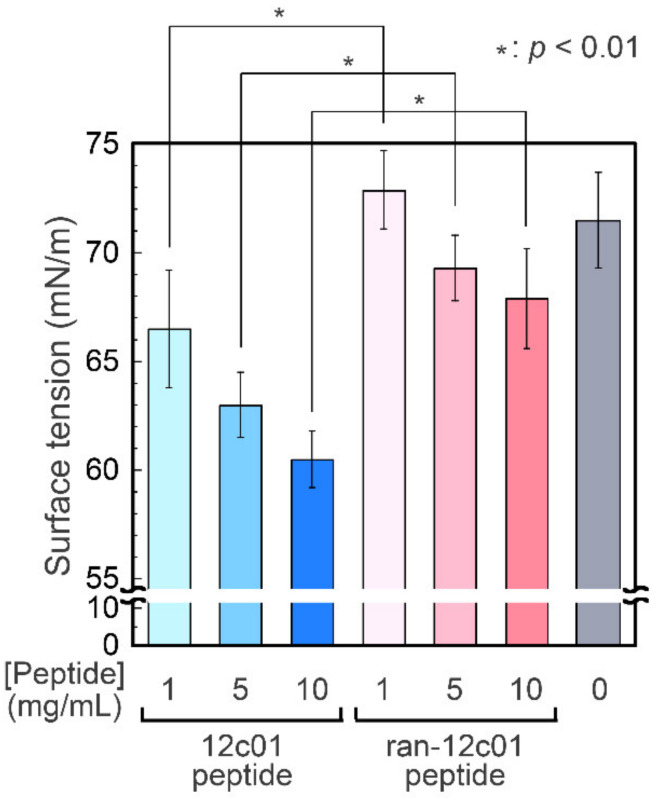
Surface tension measurements of the screened 12c01 peptide and randomized 12c01 peptide-dissolved ultrapure water. The surface tension values are average values with standard deviations from five independent measurements.

The surface tension of the peptide solutions was determined by conducting pendant drop tensiometry. The surface tension value of the 12c01 peptide solution was lower than that of ultrapure water and decreased with increasing peptide concentration (Figure 4), demonstrating certain surface activity of the 12c01 peptide. On the other hand, the ran-12c01 peptide did not lower the value of the surface tension (which was the same as that for the ultrapure water within experimental error), showing the essential role of the amino acid sequence of the 12c01 peptide on the surface activity. The surface tension values were unchanged during incubation for several hours. Therefore, the decreased surface tension was due to immediate adsorption of the 12c01 peptide onto the air–liquid interfaces. Unfortunately, although the minimum surface tension value measured in this study was 60.5 ± 1.3 mN/m (10 mg/mL for the 12c01 peptide), which is greater than that obtained for typical synthetic surfactants and biosurfactants (ranging from 20 to 60 mN/m) at the same concentrations [42], a certain surface activity of the screened peptide was observed for the first time. These results demonstrate that the 12c01 peptide can accumulate at the air–liquid interface, lowering surface tension as a surfactant, even though the peptide is freed from phages.

**Figure 5:**
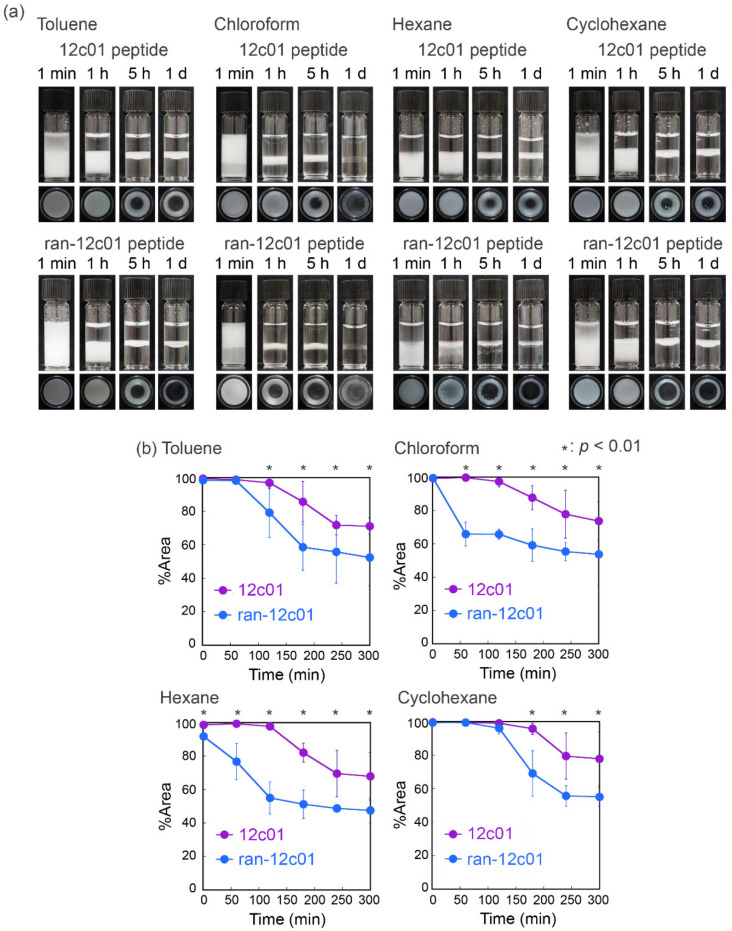
Emulsion formation using various organic solvents and peptides. (**a**) Optical photographs of emulsions prepared with the 12c01 and ran-12c01 peptides (each 1 mg/mL). (**b**) Comparison of the stability of emulsions prepared with the 12c01 and ran-12c01 peptides. The percent area values are average values with standard deviations from three independent measurements.

The capability of the 12c01 peptide as a surfactant was determined via emulsification using toluene, chloroform, hexane, and cyclohexane as organic phases (Figure 5a). Emulsions were successfully prepared with all the organic solvents used in this study and maintained for at least one day, indicating certain surface activity as surfactants. The stability of the emulsions prepared with the 12c01 peptide was compared with those prepared with the ran-12c01 peptide using the coverage areas for the emulsions (Figure 5b). In all the organic solvents, the quantity of emulsions prepared with the 12c01 peptide was greater than those prepared with the ran-12c01 peptide, demonstrating essential roles of the amino acid sequence in emulsification. The orders of stability for emulsions prepared with the 12c01 peptide and the ran-12c01 peptide were cyclohexane > chloroform > toluene ≈ hexane and cyclohexane > toluene > hexane > chloroform, respectively. Because the order of these stabilities did not appear to be related to the parameters for the organic solvents [43,44,45,46], we considered that the order was determined by the affinity of side chains of each peptide for water and organic solvents. Additionally, because the stability was different depending on the amino acid sequences, simple hydrophobicity based on amino acid compositions in the peptide did not affect emulsion formation and stability. These results indicated that the amino acid sequence of the screened 12c01 peptide acted as an emulsifier to form stable emulsions through its inherent surface activity.

## 4. Conclusions

We screened peptides from a phage-displayed peptide library with surface activity for sufficient accumulation at the air–liquid interface. Emulsification experiments indicated that a screened phage clone displaying the peptide (12c01 phage clone) showed higher surface activity compared to WT phages. When phages were used at a lower concentration (15 nM), the 12c01 phage clone formed emulsions, whereas WT phages did not. Furthermore, when a higher concentration of phages (150 nM) was used for emulsification, the formation and stability of emulsions were the same. AFM observation of the phages accumulated at the emulsion surface demonstrated different morphologies between the emulsions prepared with the 12c01 phage clone and WT phages. Additionally, when phages were used at a concentration of 150 nM for emulsification, coverage of the emulsions prepared using WT phages was smaller than that of the 12c01 phage clone immediately after emulsification. These results using phages indicate different mechanisms for assembly at the emulsion surface due to the higher surface activity of the 12c01 phage clone. A chemically synthesized peptide (12c01 peptide) was utilized for surface tension measurements. The surface tension of the 12c01 peptide-dissolved solutions decreased with increasing peptide concentration, demonstrating certain surface activity of the peptide. On the other hand, a peptide with a randomized sequence for the 12c01 peptide (ran-12c01 peptide) did not show decreased surface tension, demonstrating essential roles for the surface activity of the 12c01 peptide. Emulsion formation experiments using the peptides revealed certain surfactant-like behavior for the 12c01 peptide with various organic solvents. Our results for identifying surfactant-like peptides on the basis of phage display methods will open up an opportunity for biosurfactant-based construction of peptidyl materials in a wide range of applications.

## Figures and Tables

**Table 1:** The amino acid sequences for phage-displayed peptides and their frequency.

Clone	Frequency	Sequence
12c01	16/26	SQDIRTWNGTRS
12c02	1/26	TFYNNPTHTPSH
12c03	1/26	SGLPASAAMPLN
12c04	1/26	SPSVRNGVSPYG
12c05	1/26	QLYHPMSRGAVK
12c06	1/26	YLNNDHFGQALT
n. i.^1^	5/26	-

^1^ Not inserted.

## Supplementary Materials

The following are available online at https://www.mdpi.com/1999-4915/12/12/1442/s1: Figure S1. Comparison of percent appearances between phage clones and library phages. Figure S2. Morphological characterization of the emulsion surface. Figure S3. Circular dichroism (CD) spectra for the 12c01 peptide in phosphate buffer or a mixed solution of phosphate buffer and trifluoroethanol (TFE). Figure S4: Structures for the peptides obtained from a molecular mechanics (MM) calculation.
