# Supplementary material for: Discovery of Surfactant-Like Peptides from a Phage-Displayed Peptide Library
Source: Viruses. 2020 Dec 15;12(12):1442. doi: 10.3390/v12121442 (PMC7765448; doi:10.3390/v12121442)
Supplement: Supplementary file 1 [file viruses-12-01442-s001.pdf]

Electronic Supplementary Information

Discovery of Surfactant-Like Peptides from a Phage-Displayed Peptide Library

Toshiki Sawada,[1,2,*] Rina Oyama,[1] Michihiro Tanaka,[1] and Takeshi Serizawa[1,*]

[1] *Department of Chemical Science and Engineering, School of Materials and Chemical Technology, Tokyo Institute of Technology*
2-12-1 Ookayama, Meguro-ku, Tokyo 152-8550, Japan

[2] *Precursory Research for Embryonic Science and Technology (PRESTO), Japan Science and Technology Agency (JST)*
4-1-8 Honcho, Kawaguchi-shi, Saitama 332-0012, Japan

E-mail: sawada@mac.titech.ac.jp (To.S.); serizawa@mac.titech.ac.jp(Ta.S.)

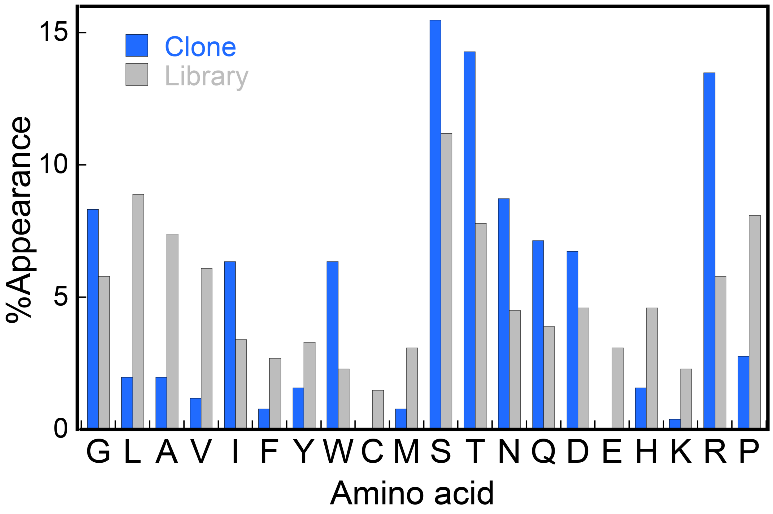

Figure S1. Comparison of percent appearances between phage clones and library phages.

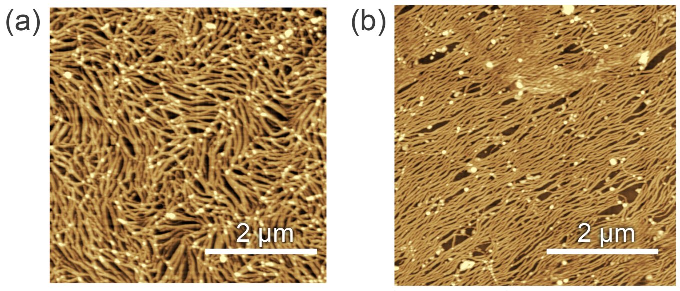

Figure 2. Morphological characterization of the emulsion surface. (a) Emulsions prepared with the 12c01 phage clone and (b) wild type phages observed after 48 hours.

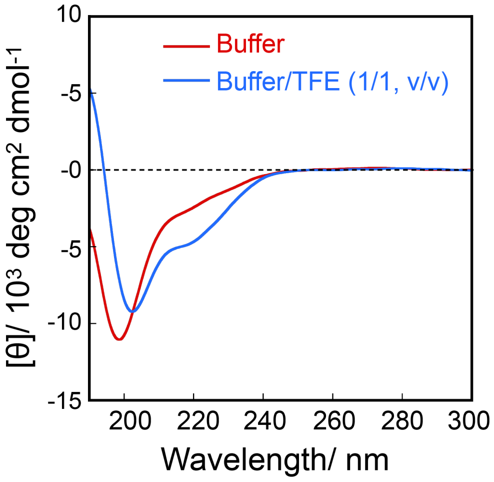

Figure S3. Circular dichroism spectra for the 12c01 peptide in phosphate buffer or a mixed solution of phosphate buffer and trifluoroethanol (TFE).

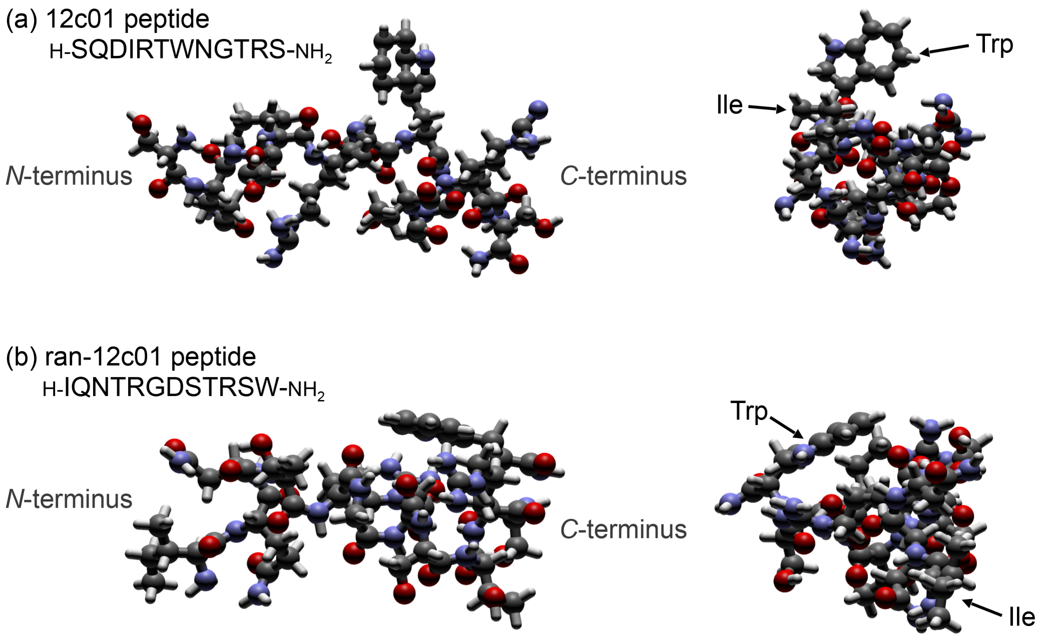

Figure S4. Structures for the (a) 12c01 peptide and (b) ran-12c01 peptide obtained from an MM calculation.
